# Retraction: Advancing enterprise risk management with deep learning: A predictive approach using the XGBoost-CNN-BiLSTM model

**DOI:** 10.1371/journal.pone.0343285

**Published:** 2026-02-23

**Authors:** The PLOS One Editors

The *PLOS One* Editors retract this article [1] because it was identified as one of a series of submissions for which we have concerns about compromised peer review.

All authors either did not respond directly or could not be reached.
